# Orlistat-Induced Gut Microbiota Modification in Obese Mice

**DOI:** 10.1155/2020/9818349

**Published:** 2020-04-08

**Authors:** Jing Ke, Yaxin An, Bin Cao, Jianan Lang, Nannan Wu, Dong Zhao

**Affiliations:** Beijing Key Laboratory of Diabetes Research and Care, Center for Endocrine Metabolism and Immune Diseases, Lu He Hospital, Capital Medical University, Beijing, China

## Abstract

**Introduction:**

Accumulating evidence has indicated that alterations of gut microbiota have been involved in various metabolic diseases. Orlistat, a reversible inhibitor of pancreatic and gastric lipase, has beneficial effects on weight loss and metabolism. However, the effect of orlistat on the composition of gut microbiota remains unclear.

**Objective:**

We aimed to explore the effect of orlistat on gut microbiota in high-fat diet (HFD) fed C57BL/6J obese mice.

**Methods:**

C57BL/6J mice were randomly divided into three groups: control (NCD), HFD, and HFD + orlistat (ORL). Mice in the NCD group were fed chow diet, while the other groups were fed HFD for 6 months, and orlistat was added in the final 3 months in the HFD + ORL group. After sacrifice, body weight and metabolic parameters were assessed, and the gut microbial composition was analyzed by 16S rRNA gene sequencing.

**Results:**

Orlistat treatment exerted beneficial effects on body weight, plasma cholesterol, and glucose tolerance. Meanwhile, orlistat treatment modified the gut microbiota, presenting as reduced total microbial abundance and obvious upregulated bacteria. Moreover, the upregulated bacteria correlated with several metabolic pathways.

**Conclusions:**

Orlistat may exert beneficial effects on body weight and glucose tolerance through modifying the composition of gut microbiota.

## 1. Introduction

Over the past decades, the prevalence of obesity has been substantially increasing worldwide with over 2.1 billion people considered to be overweight or obese [1]. Overweight and obesity are frequently associated with greater risk of developing various chronic diseases, including type 2 diabetes mellitus (T2DM) [2], cardiovascular disease [3], nonalcoholic fatty liver disease [4], musculoskeletal disorders (mainly osteoarthritis) [5, 6], and even certain types of cancers [7]. Given the boosted socioeconomic expenditure and increased health burden, overweight and obesity have been considered to be an enormous challenge in many countries nowadays.

Expanding evidence has shown that gut microbiota, as a key environmental factor, was involved in the development of obesity and metabolic disorders. In human studies, reduced microbial gene richness [8, 9] and bacterial diversity [10] have been reported in obese subjects. In addition, the causative role of gut microbiota in obesity has been confirmed in the animal experiments by fecal transplantation in germ-free mice [11, 12]. Proposed mechanisms accounting for the causative link involve the roles of gut microbiota in elevation in energy production from diet [12], modulation of host metabolism [13], and contribution to chronic low-grade inflammation [14].

Orlistat (ORL), a reversible gastric and pancreatic lipase inhibitor, is widely available in more than 120 countries worldwide. It is reported that orlistat blocks hydrolysis of triglycerides and reduces absorption of ingested fat by about 30% [15]. Given the good safety profile demonstrated by a 4-year longitudinal study [16], orlistat has been approved for long-term use to manage body weight in obese and overweight adults and also adolescents [17].

To the best of our knowledge, there are limited studies focusing on the effects of orlistat on gut microbiota. Therefore, our study aims to explore the effects of orlistat on gut microbiota in high-fat diet (HFD) induced obese mice.

## 2. Materials and Methods

### 2.1. Animals and Treatments

Wild type male C57BL/6J mice, supplied by Beijing Vital River Laboratory Animal Technology Co., Ltd., were housed in groups of 2-3 in the temperature-controlled breeding room with a 12 h light/dark cycle. Food and water were provided ad libitum. After acclimatization, mice were randomly allocated to three groups as follows: control (NCD), HFD, and HFD + ORL. NCD mice received normal chow diet (4.5% fat and 0.02% cholesterol), whereas other mice were fed HFD (34% fat and 1% cholesterol, Catalog no. D12492 mod, BioServices, the Netherlands) for 6 months aggregately. Moreover, mice in the HFD + ORL group were additionally supplemented with orlistat (50 mg/kg, Beijing Keao Xieli Company) in the final 3 months.

By the end of 6-month experiments, blood samples were collected from anesthetized mice by an intraperitoneal injection of pentobarbital sodium, centrifuged at 3000 rpm for 8 min, and then stored at −20°C prior to utilization following an overnight fast. They were sacrificed directly after the collection of blood samples. Fecal pellets of each mouse were collected in sealed containers and immediately stored at −80°C with prior snap-freezing in liquid nitrogen until DNA extraction. All experiments were approved by the competent Institutional Review Boards of Capital Medical University.

### 2.2. Metabolic Assessments

For the intraperitoneal glucose tolerance test (IPGTT), a baseline glucose level at 0 min was obtained from the tip of the tail vein in mice using a glucometer (Accu-Chek, Roche) after an 8-hour fast. Blood glucose concentrations were then determined at 15, 30, 60, 90, and 120 min following an intraperitoneal injection with 10% glucose at 10 *μ*l/g of body weight in each mouse. The corresponding data of area under the curve (AUC) were then obtained. Serum cholesterol levels were quantified (BioSino Biotechnology, Beijing, China).

### 2.3. DNA Extraction and Amplicon Generation

Genomic DNA was isolated from approximately 100 mg of feces using the NucleoSpin Soil kit (MACHEREY-NAGEL, Germany) following the manufacturer's instruction.

The universal primer set, 515F (5′-GTG CCA GCM GCC GCG GTA A-3′) and 806R (5′-GGA CTA CNN GGG TAT CTA AT-3′), was used for the amplification of the V3-V4 region of bacterial 16S rRNA gene. The PCR reaction was carried out as follows: initial denaturation at 98°C for 1 min, followed by 30 cycles of denaturation at 98°C for 10 s, annealing at 50°C for 30 s, elongation at 72°C for 30 s, and finally, 72°C for 5 min.

### 2.4. PCR Products

Mix the same volume of 1x loading buffer (containing SYBR green) with PCR products and operate electrophoresis on 2% agarose gel for detection. Samples with bright main strips between 400 and 450 bp were chosen for further analysis. PCR products were mixed in equidensity ratios. Then, the mixture of PCR products was purified with GeneJET Gel Extraction Kit (Thermo Scientific).

### 2.5. Library Preparation and Sequencing

The analysis of bacterial community structure was performed by 16S rRNA gene sequencing in the V3-V4 region via the Illumina HiSeq platform.

Sequencing libraries were generated using NEB Next® Ultra™ DNA Library Prep Kit for Illumina (NEB, USA), and index codes were added. The library quality was evaluated on the Qubit@ 2.0 Fluorometer (Thermo Scientific) and Agilent Bioanalyzer 2100 system. In the end, the library was sequenced on an Illumina HiSeq 2500 platform. Then paired-end reads with 250 bp were generated.

### 2.6. Data Analysis of 16S rRNA Gene Sequencing

Based on samples' unique barcode, raw reads were allocated to different samples, then the paired-end reads from the original DNA fragments were merged to raw tags by using Vsearch software (Version 2.10.4) when there were overlaps between reads1 and reads2. Then the merged raw tags were filtered and developed into clean tags according to Vsearch quality-controlled process. After the quality control, the chimera sequence was detected and discarded by USEARCH (version 10.0.240) using the de_novo method, and the remaining sequence was aligned to Silva database to further discard chimera sequence. Finally, non-chimera clean tags were developed and defined as effective tags. Sequences with ≥97% similarity were clustered into the same operational taxonomic units (OTUs). We picked representative sequences for each OTU and used the GreenGene database (Release gg_13_5) to annotate taxonomic information for each representative sequence. A representative sequence from each OUT was selected and annotated taxonomy by using GreenGene database (Release gg_13_5).

#### 2.6.1. Alpha and Beta Diversity

To compute alpha diversity (within-sample), the OTU table was rarified, and richness index was calculated based on the genera profile of NCD, HFD, and HFD + ORL groups. Then, for beta diversity (between-sample), the OTU table was used to generate weighted UniFrac distance matrix, and principal coordinate analysis (PCoA) was performed and displayed by ggplot2 package in R (Version 3.5.2).

#### 2.6.2. Taxonomic Discovery Analysis

We analyzed significant differences in the relative abundance of taxa among three groups by using linear discriminant analysis effect size (LEfSe) [18]. Taxa with value from linear discriminant analysis (LDA) more than 2 at a *p* value <0.05 were considered significantly enriched.

#### 2.6.3. Co-occurrence Network Analysis

Co-occurrence network analysis was conducted independently on each group. OTUs with 9 highest relative abundances of the microbiota were combined based on the lowest common taxonomy assignments down to genus level and then were subjected to Spearman correlation analysis of their occurrence patterns, using the non-rarified sequence data. An edge was set between two bacterial genera if the *p* < 0.05 and |*r*| was >0.7 and further visualized through network analysis with R (Version 3.5.2).

#### 2.6.4. Functional Metagenome Predictions

In terms of functional metagenome prediction, OTU representative sequences were captured from GreenGene database by using the USEARCH global alignment command. We executed reconstruction of metagenome by using PICRUSt [19]. Accuracy of the predicted metagenomes was evaluated by determination of the nearest sequenced taxon index. Predicted functional genes were classified into Kyoto Encyclopedia of Genes and Genome (KEGG) and orthology (KO) and compared three groups using STAMP [20]. To explore the relationship between enriched taxa and significant functional metagenomes, Spearman's correlation coefficients were calculated.

### 2.7. Statistical Analysis

One-way analysis of variance (ANOVA) with post hoc Tukey HSD test was conducted to detect differences in metabolic indices as well as abundance of genera and KOs. *p* values were adjusted in multiple testing followed by the Benjamini–Hochberg correction. Adjusted *p* < 0.05 was considered significant.

## 3. Results

### 3.1. Effects of Orlistat on Weight, Lipid, and Glucose Tolerance

**Figure 1:**
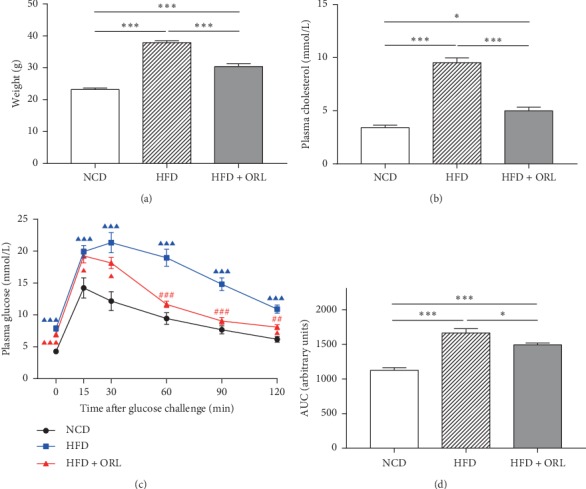
Effects of orlistat on weight, cholesterol, and glucose tolerance in HFD-induced obese mice. (a) Weight, (b) serum cholesterol, (c) IPGTT, and (d) AUC analysis of the IPGTT plot in mice of three groups. All data are expressed as mean ± SEM. ^▲▲▲^*p* < 0.001, ^▲▲^*p* < 0.01, ^▲^*p* < 0.05 compared to NCD mice and ^###^*p* < 0.001, ^##^*p* < 0.01, ^#^*p* < 0.05 compared with the HFD group.

The effects of orlistat on metabolic parameters were examined. As expected, mice in the HFD group significantly gained weight (37.9 ± 0.6 versus 23.2 ± 0.5, respectively, *p* < 0.0001) than in the NCD group, whereas mice in the HFD + ORL group showed markedly reduced body weight compared with those in the HFD group (30.4 ± 0.9 versus 37.9 ± 0.6, *p* < 0.0001) (Figure 1(a)). Compared to NCD, mice in the HFD group demonstrated increased serum cholesterol (9.5 ± 0.4 versus 3.4 ± 0.2, *p* < 0.0001), while orlistat treatment significantly reserved the changes induced by HFD (5.0 ± 0.3 versus 9.5 ± 0.4, *p* < 0.0001) (Figure 1(b)). In terms of glucose tolerance, fasting and postload plasma glucose levels were significantly higher in obese mice induced by HFD than in NCD group. Among the obese mice, the HFD + ORL group showed marked improvements in postload glucose levels and correspondingly lower area under the curve than in the HFD group (Figures 1(c) and 1(d)).

### 3.2. The Modification in the Composition of Gut Microbiota after Orlistat Treatment

A total of 5783438 valid sequences were generated from 79 fecal samples (19 samples from the NCD group, 35 in the HFD group, and 25 from the HFD + ORL group). After data trimming and quality filtering, 5454588 high-quality sequences (representing ∼94% of the total sequences) were required, with an average of 69045 per sample.

**Figure 2:**
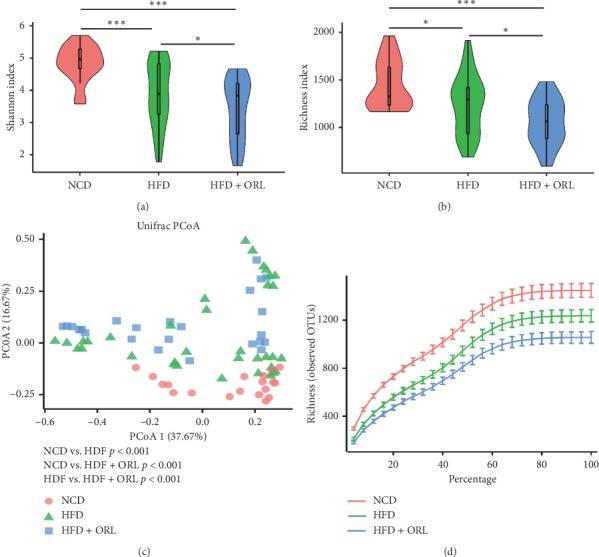
Effects of orlistat on gut microbial composition in HFD-induced obese mice. Pairwise comparisons of *α* diversity, including (a) Shannon index and (b) Richness index, were detected among the NCD, HFD, and HFD + ORL group (blue squares). (c) Analysis of *β* diversity by principal coordinates analysis (PCoA) of weighted Unifrac distances in NCD (dots in orange), HFD (green triangles), and obese mice with orlistat supplementation (blue squares). (d) Rarefaction curves according to species richness (observed OTUs) in three groups. As the curves tend to be flat, enough extracted sequences were detected.

Mice fed HFD, particularly those supplemented with orlistat, showed decreased microbial diversity and richness demonstrated by both Richness index (Figure 2(a)) and Shannon index (Figure 2(b)). Rarefaction curves from the three groups reaching the plateau presented sufficient sequencing depth of all fecal samples (Figure 2(d)). Moreover, similar to Figure 2(b), rarefaction curves indicated the observed OTUs were lower in mice of the HFD + ORL group than those in the NCD and HFD groups (*p* < 0.001 and *p* = 0.039, respectively) (Figure 2(d)). The results suggested that HFD decreased microbial diversity and richness, which could be less induced by treatment with orlistat. Concerning beta diversity of microbiome, distinct heterogeneous structures of bacterial community were displayed among the three groups (Figure 2(c)).

**Figure 3:**
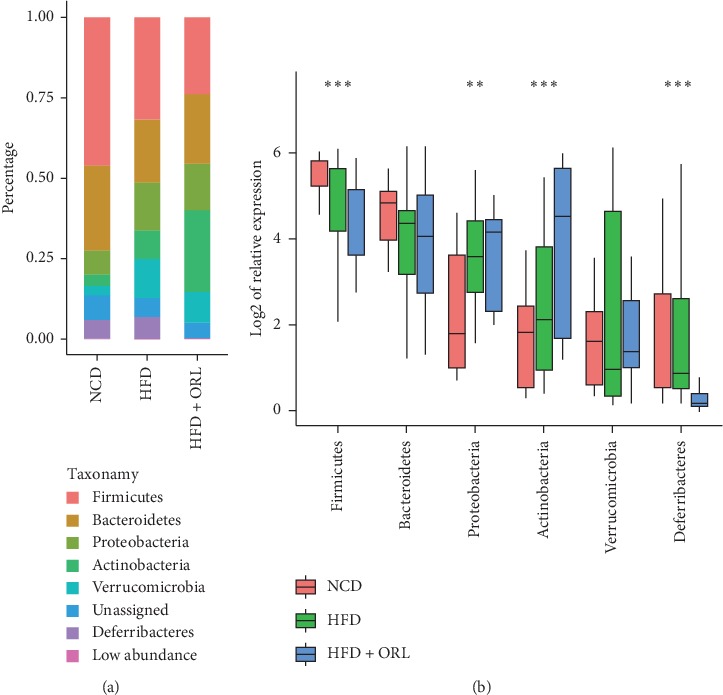
Relative abundances of bacterial community at different levels among 3 groups. (a) The bacterium with the top 6 relative abundances among the three groups at phylum level. (b) The boxplot presents comparisons of the 6 highest bacteria at phylum level among the three groups using one-way ANOVA with Tukey HSD test. ^*∗∗∗*^*p* < 0.001, ^*∗∗*^*p* < 0.01, and ^*∗*^*p* < 0.05.

To compare the microbial composition of fecal samples in the three groups, the bacterium with the top 6 relative abundances among the three groups at the phylum level was shown in a stackplot (Figure 3(a)). At phylum level, the proportions of Firmicutes and Deferribacteres were decreased significantly in mice fed HFD and were further reduced in those treated with orlistat. Actinobacteria and Proteobacteria were more abundant in HFD + ORL mice than mice in NCD and HFD groups (Figure 3(b)).

**Figure 4:**
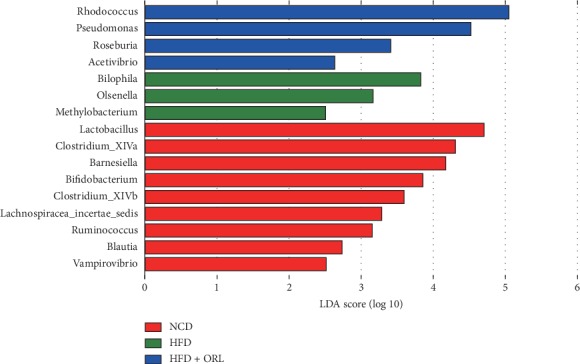
Differentially abundant bacterial taxa among the groups. Enriched bacterial taxa at genus level were identified by linear discriminant analysis (LDA) with effect size measurements shown in a log scale among three mice groups: the NCD (red histograms), HFD (green histograms), and HFD+ORL group (blue histograms).

Specific bacterial biomarkers, whose relative abundance differed markedly among the three groups, were identified using the LDA effect size method and shown by the histograms in Figure 4. The results revealed 16 significantly different genera, among whom *Methylobacterium*, *Olsenella*, and *Bilophila* were relatively higher in mice from HFD samples, whereas *Barnesiella*, *Bifidobacterium*, *Blautia*, *Clostridium XlVa*, *Clostridium XlVb*, *Lachnospiraceae incertae sedis*, *Lactobacillus*, *Ruminococcus*, and *Vampirovibrio* were more enriched in NCD mice. The HFD + ORL group showed distinct microbial structure, which was enriched in *Pseudomonas*, *Rhodococcus*, *Roseburia*, and *Acetivibrio* at genus level.

### 3.3. Co-occurrence Networks of Gut Microbiota

**Figure 5:**
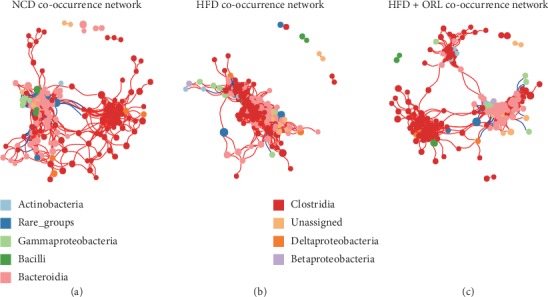
Bacterial co-occurrence network analysis associated with high-fat diet and orlistat treatment. Each node in the network represents one of the 9 highest bacteria and the size of each node represents its relative abundance. Edges indicate the bacterial connections expressed Spearman's correlation coefficients with values greater than cut-off of 0.7 and adjusted *p* value less than 0.05. Positive and negative correlations are shown in red and blue edges, respectively. (a) NCD co-occurrence network. (b) HFD co-occurrence network. (c) HFD + ORL co-occurrence network

To evaluate the bacterial ecosystem structure induced by HFD and orlistat treatment, we explored the co-occurrence patterns of microbial communities at phylum level based on Spearman's rank correlations (Figure 5). A relatively complex network of correlations between bacteria was observed in both NCD and HFD + ORL groups, while a comparatively simplified network was presented in the HFD group.

As shown in Figure 5, the average path length (APL) between nodes was 3.45, 2.50, and 3.90 edges, respectively, in the NCD, HFD, and HFD + ORL network. However, mice in the NCD and HFD + ORL groups displayed decreased density of bacterial correlation network and reduced number of neighbors in comparison with the HFD group. These results indicated that HFD was associated with alterations of gut microbial communities, and orlistat treatment may be prone to modify the microbial structure from “high-fat” module to “healthy” module.

### 3.4. Orlistat Promotes Functional Shifts in the Gut Microbiota

**Figure 6:**
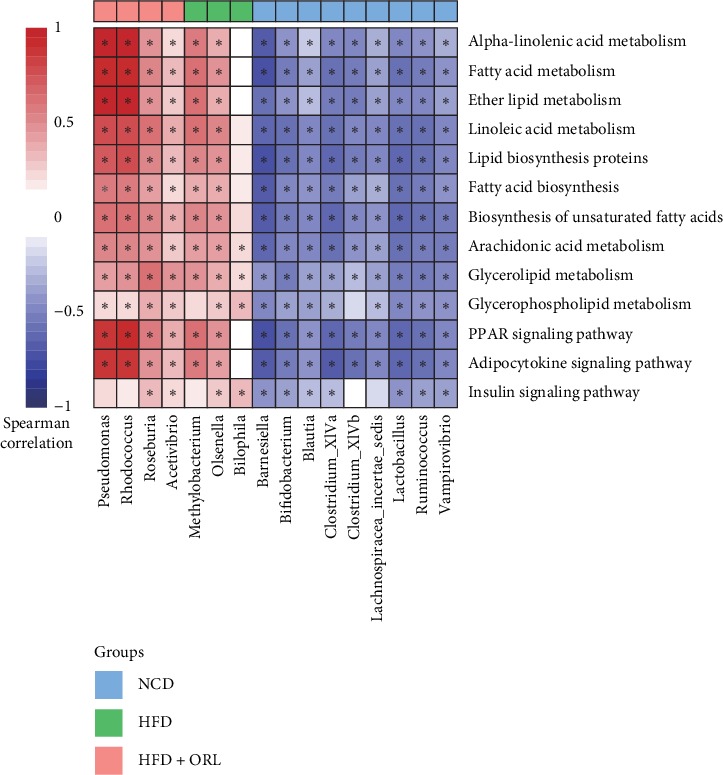
Heatmap of Spearman's correlation between enriched bacterial taxa and third level of KEGG metabolic pathways according to clustering of three groups. Spearman's correlation coefficients were calculated and shown in the left, with red representing more positively and blue being more negatively correlation. The clustering of the NCD (light blue box), HFD (green box), and HFD + ORL (orange box) group was presented above the figure. ^*∗*^Spearman's correlation is significant with *p* < 0.05.

To further investigate functional changes in the gut microbiome after orlistat treatment, we annotated genes to KOs. We identified 41 active secondary KEGG pathways, of which 33 were significantly different in abundance among the three groups (*p* < 0.05). Furthermore, we observed that “lipid metabolism” and “endocrine system” pathways, which are reported to be involved in lipid and carbohydrate metabolism. The results found that the above two secondary pathways were highly enriched in the HFD + ORL group in comparison with the NCD and HFD groups (Supplemental Figures 1A and 1B). Then we analyzed the correlation between gut bacteria and metabolism pathways. In the NCD group, bacterial biomarkers inversely correlated with lipid and carbohydrate metabolism. In the HDF group, *Methylobacterium* and *Olsenella* were moderately related to the pathways mentioned above. In contrast, the HFD + ORL group showed pronounced enrichments in the following pathways with altered microbiomes. More specifically, *Rhodococcus* and *Pseudomonas* in the HFD + ORL group were highly positively correlated with metabolic pathways, including alpha-linolenic acid metabolism, fatty acid metabolism, ether lipid metabolism, PPAR signaling pathway, and adipocytokine signaling pathway (Figure 6).

## 4. Discussion

As an anti-obesity medicine, multiple clinical studies have proved the effects of orlistat on weight loss and glycemic improvements [21–24]. Sahebkar and colleagues [22] have investigated the efficacy of orlistat on body weight in a meta-analysis of 33 studies involving 9732 participants. The data showed that orlistat was associated with a slight but significant decrease in body weight. Besides, the other three studies documented the efficacy of orlistat on glucose improvements and possible biochemical conversion to type 2 diabetes [21, 23, 24]. In our study, chronic HFD consumption resulted in weight gain and impaired glucose tolerance, whereas treatment with orlistat decreased body weight significantly and improved the glucose tolerance than mice in HFD group. It is known that lipase inhibition was the primary mechanism of orlistat in reduction of body weight and possible improvements in metabolism. Our study confirmed the positive effects of orlistat on weight loss and glucose tolerance.

Although a previous study by Jiao et al. demonstrated the antiobesity effect of blueberry polyphenols extract by modulation of gut microbiota, utilized orlistat as control group, and focused on lipid metabolism [25], in our study, we examined the effect of orlistat, a classic anti-obesity medication, on metabolic alleviation, in particularly, in impaired glucose metabolism, and gut microbiota, which was analyzed more comprehensively. Our results showed that HFD consumption caused significantly decreased microbial diversity and richness, and orlistat supplementation further reduced it. Several studies investigating the anti-obesity effects of natural active ingredients, such as catechin and blueberry polyphenols extract [25, 26], observed the modulation of the gut microbiota composition with downregulation of the microbial diversity. Similar results on orlistat were also documented [25]. Moreover, a previous study by Collins et al. showed a decrease in observed species in HFD fed mice compared to a low-fat group; reversion of such changes was not found in group supplemented with polyphenols [27]. Another explanation may account for the adverse events related to gastrointestinal tract which results from the minimal fat absorption, such as oily spotting, fecal urgency, oily stool, diarrhea, and flatus with discharge. Additionally, our results demonstrated that Actinobacteria and Proteobacteria were more abundant in HFD + ORL mice than other groups. Moreover, recent studies have shown that several medications affecting body weight could modify gut microbiota. Treatment with metformin could directly regulate gut microbiota, especially those who affected pathways of metal homeostasis [28, 29]. Diabetic patients receiving acarbose exhibited changes in gut microbiota composition and related microbial genes which are involved in bile acid metabolism and also showed different treatment response associated with gut microbiota prior to treatment [30]. Furthermore, liraglutide resulted in more lean-related microbial phenotypes [31, 32].

Regarding the changes in microbial composition in human and mice responding to obesity, numerous studies have reported that several phyla of microbiota are associated with obesity. Firmicutes, Bacteroidetes, and Actinobacteria, the most abundant phylum in the gut microbiota, have been documented to be associated with obesity. Obesity or HFD consumption has been found to be linked with a decrease in Bacteroidetes, which exerts immunomodulatory effects on host and a higher abundance of Firmicutes that play a role in energy resorption and obesity. Other studies have demonstrated higher abundance of Actinobacteria in obese subjects [10]. These findings are mostly in line with our results. In our study, the ratio of Firmicutes to Bacteroidetes was slightly reduced after HFD feeding and further decreased with orlistat treatment. Previous studies have suggested that obesity or HFD consumption resulted in an increased ratio of Firmicutes to Bacteroidetes.

Further analysis found that mice in the HFD + ORL group showed obvious enrichment in genes involved in the endocrine and lipid metabolism. More specifically, *Rhodococcus* and *Pseudomonas* were highly correlated with the alpha-linolenic acid (ALA) metabolism, fatty acid metabolism, ether lipid metabolism, PPAR signaling pathway, and adipocytokine signaling pathway. ALA, an *n*-3 polyunsaturated fatty acid, is one of the essential fatty acids. Gut microbiota plays an important role in the metabolism of ALA to conjugated linolenic acid [33], which was documented to have antiadipogenic effects in several studies [34]. Another KEGG pathway with significant enrichment in the HFD + ORL group was PPAR (peroxisome proliferator-activated receptors) signaling pathway, which plays a crucial role in adipogenesis for obesity development [35]. PPAR are members of the nuclear receptor (NR) superfamily of ligand-dependent transcription factors. PPARs can function as lipid sensors and regulate differentiation and metabolism of adipocytes. Defects in PPARs have been observed in obesity and insulin resistance, and activation of PPARs, which plays a crucial role in various homeostatic processes involving metabolism of carbohydrates, protein, and lipids, could ameliorate inflammation and fat accumulation in the adipose tissue [36]. Moreover, we also observed increased adipocytokine signaling pathway in the HFD + ORL group. Adipocytokines, derived from adipose tissue, can function as hormones (such as leptin, adiponectin, and vistafin) and cytokines (such as IL-6 and TNF-alpha) to regulate energy homeostasis and mediate inflammation and immunity [37]. These observations are in line with our results and may explain the possible mechanisms underlying weight loss, glycemic, and lipid improvements. In our study, we found that orlistat treatment could modify the enrichment of some particular bacterium, which is associated with glucose, lipid metabolism, and inflammation pathway. The specific relationships between the microbiota variation and metabolism still needs further study for illumination.

In summary, the present study suggested that orlistat may exert beneficial effects on body weight and glucose metabolism by modifying gut microbiota, which may offer a novel mechanism of orlistat. However, the more specific mechanisms of orlistat on gut microbiota and its related pathways need further research.

## Data Availability

Currently, the data are unavailable, since the original high-throughput data were considerably large and we had no experience to upload such large data. If needed, we could upload as required.

## Supplementary Materials

Supplementary Materials Supplementary Figure 1: Predicted metagenomic function related to secondary KEGG pathways. Enriched KEGG pathways: (A) “endocrine system” and (B) “lipid metabolism” were detected by PICRUSt and STAP. Pairwise comparisons among HFD + ORL (green box), HFD (orange box), and NCD group (blue box) were performed. ^*∗∗∗*^*p* < 0.001.
