# Supplementary material for: Orlistat-Induced Gut Microbiota Modification in Obese Mice
Source: Evid Based Complement Alternat Med. 2020 Apr 8;2020:9818349. doi: 10.1155/2020/9818349 (PMC7168719; doi:10.1155/2020/9818349)
Supplement: Supplementary Materials — Supplementary Figure 1: Predicted metagenomic function related to secondary KEGG pathways. Enriched KEGG pathways: (A) “endocrine system” and (B) “lipid metabolism” were detected by PICRUSt and STAP. Pairwise comparisons among HFD + ORL (green box), HFD (orange box), and NCD group (blue box) were performed. ∗∗∗p < 0.001. [file 9818349.f1.pdf]

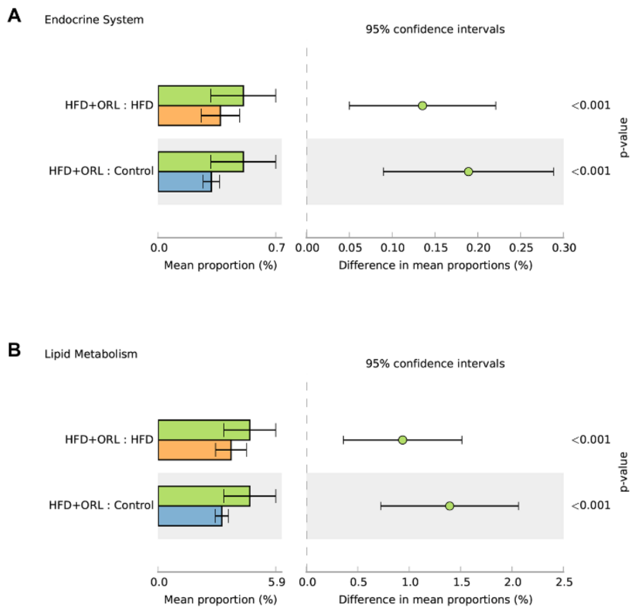

Suppl. Fig. 1. Predicted metagenomic function related to secondary KEGG pathways. Enriched KEGG pathways, (A) “Endocrine system” and (B) “Lipid metabolism” were detected by PICRUSt and STAMP. For pairwise comparison among HFD+ORL (green box), HFD (orange box) and control group (blue box), mean proportions (%) and differences in mean proportions (%) were presented at left and right, respectively. *P* values were also shown for each comparison.
